# Acute changes in antioxidants and oxidative stress to vigorous arm exercise: an intervention trial in persons with spinal cord injury and healthy controls

**DOI:** 10.1038/s41394-023-00590-6

**Published:** 2023-07-13

**Authors:** Matthijs F. Wouda, Hanne Bjørg Slettahjell, Eivind Lundgaard, Nasser E. Bastani, Truls Raastad, Rune Blomhoff, Emil Kostovski

**Affiliations:** 1 Sunnaas Rehabilitation Hospital, Research department, Nesoddtangen, Norway; 2 University of Oslo, Department of Nutrition, Institute of Basic Medical Sciences, Oslo, Norway; 3 Norwegian School of Sport Sciences, Department of Physical Performance, Oslo, Norway; 4 Oslo University Hospital, Department of Clinical Service, Division of Cancer Medicine, Oslo, Norway; 5 Stiftelsen Manifestsenteret, Røyken, Norway

**Keywords:** Physiology, Biomarkers

## Abstract

**Study design:**

Intervention trial.

**Background:**

Literature remains unclear on possible health benefits and risks assosciated with high intensity exercise for persons with SCI. Elevated oxidative stress levels might influence their ability to exercise at high intensity. We investigated several biomarkers of oxidative stress and antioxidant defense at rest, during and after vigorous exercise among persons with chronic SCI.

**Setting:**

Sunnaas Rehabilitation Hospital, Norway.

**Methods:**

Six participants (five males) with chronic SCI (AIS A, injury level thoracic 2–8, >1 year postinjury) and six matched able-bodied controls performed two maximal arm-cranking tests, with one-three days in between. During the second exercise test, participants performed three bouts with four minutes arm cranking at high intensity (85–95% of peak heart rate (HR_peak_)), before they reached maximal effort. Blood and urine biomarkers for oxidative stress and antioxidant levels were collected at six time points at the day of the second exercise test; baseline, at high intensity exercise, at maximal effort, at five, 30 and 60 min post-exercise, and 24 h post exercise.

**Results:**

Participants with SCI had significant lower levels of creatinine (∆16 µmol/L, *p* = 0.03), α-carotene (∆0.14 nmol/L, *p* < 0.001) and β-carotene (∆0.51 nmol/L, *p* = 0.001) at baseline compared to controls. Urine and blood biomarkers of oxidative stress and antioxidant levels showed similar response to vigorous exercise in the SCI and control group.

**Conclusions:**

SCI participants showed similar changes in redox status during high intensity exercise compared to matched able-bodied. SCI participants had lower levels of exogen antioxidants both before, during and after vigorous exercise.

## Introduction

Individuals with spinal cord injury (SCI) have a distinctive physiology, characterized by a decrease in lean body mass, adipose tissue accumulation, increased risk of osteoporosis, anabolic deficiency, autonomic dysfunction, and cardiometabolic disease [1, 2]. Muscle mass depletion and intramuscular fat infiltration after SCI reduces peripheral glucose utilization and muscle mitochondrial content [3]. Moreover, skeletal muscle fibers below the SCI lesion are converted from oxidative to fast glycolytic type fibers and results in muscles that are highly fatigueable and prone to exercise-induced damage [4]. Aging, as well as obesity and diabetes type 2, is generally linked to impaired mitochondrial activity [5, 6]. Many of these consequences of SCI may lead to oxidative stress. In particular, functional decline of the electron transport chain and reduced mitochondrial respiration in affected muscles, may cause elevated levels of oxidative stress [7, 8].

Redox status is a physiological equilibrium in human cells, in which oxidants and antioxidants constantly interact. Oxidative stress is a condition where the body’s antioxidant defense cannot attenuate the damaging effects of free radicals and other reactive oxygen species (ROS). Exercise and repetitive muscle contractions produces “normal” levels of ROS which is essential for normal cellular homeostasis and function. However, overproduction of ROS induced by exhaustive high-intensity exercise, may result in oxidative stress causing cellular damage and skeletal muscle atrophy [9]. 8-Oxo-2’-deoxyguanosine (8-oxo-dG) is a major product of DNA oxidation and level of 8-oxo-dG in urine is a well-established biomarker of oxidative stress [10].

Spinal cord injury forces an extreme form of physical inactivity on the denervated muscle, and excessive ROS production is also demonstrated in prolonged periods of skeletal muscle inactivity [11]. The combination of immobilized, atrophied muscle after SCI and increased exercise load of remaining functional muscle groups, may easily result in overproduction of ROS after SCI. Thus a well-functioning antioxidative system is imperative to diminish oxidative stress. Endogeneous antioxidants include ROS-specific antioxidant enzymes that are expressed in skeletal muscle: SOD (Superoxide dismutase), catalase and glutathione peroxidase (GPx), while important non-enzyme exogeneous antioxidants include vitamin E (tocopherols and tocotrienols), vitamin C, thiols (glutathione, thioredoxin, α-lipoic acid), polyphenols (flavonoids, tannins) and carotenoids. In a study carried out by Bastani et al. (2012), participants with SCI had reduced levels of plasma antioxidants and increased markers of oxidative stress in the acute phase (1 month after injury) compared to able-bodied controls [7]. Although there was some improvement over time, the participants with SCI still had lower levels of antioxidants in plasma up to one year after injury.

Physical activity and exercise is important after SCI to minimize a sedentary lifestyle, promote independent mobility and counteract the negative health issues related to SCI [12]. Exercise guidelines for persons with SCI have been developed [13], however questions regarding optimal intensity, duration and frequency in relationship to health and secondary effects of strenuous training remains largely unanswered [14]. There is a concern that high intensity exercise may cause unhealthy levels of oxidative stress after SCI due to reduced antioxidant capacity, and thereby negatively impact overall health and restitution of functional muscle groups [14]. Consequently, understanding potential confounding effects of oxidative stress associated with exercise after SCI is important to ensure optimal recovery and exercise recommendations through aging with SCI.

The aim of this study was to measure biomarkers of oxidative stress and antioxidants, at rest, during and after a single bout of high-intensity arm cranking exercise in persons with chronic SCI (>1 year postinjury). We included a matched able-bodied control group to compare the changes in biomarkers. Our hypothesis was that persons with chronic SCI have a lower redox capacity in response to a HIIT exercise compared to able-bodied individuals.

## Methods

### Design

Intervention trial in persons with chronic spinal cord injury and healthy controls.

### Participants

Between February 2016 until April 2018, six persons with chronic (>1 year post injury) SCI (*n* = 6), who received inpatient rehabilitation at Sunnaas Rehabilitation Hospital were included. Wheelchair dependent persons with chronic SCI (>1 year post injury) were contacted for inclusion if they were aged between 18 and 70 years, had traumatic or non-traumatic complete SCI (AIS A) [15], with a lesion level below the second thoracic vertebra, i.e. intact function in upper arms. In addition, healthy controls (*n* = 6) were recruited among employees at the hospital in the same time period. A case-control matching procedure was used to match SCI cases and controls based on age, sex and Body Mass Index (BMI).

The participants with SCI needed permission from their medical doctor to be included to the study. Participants were excluded if they had significant concurrent medical conditions that limit their physical capacity, e.g., psychiatric conditions, orthopedic diseases, or uncontrolled cardiopulmonary disease.

### Exercise tests

All participants performed two exercise tests, on two separate days, with 1–3 days in between. First, a maximal arm cranking test was performed to define the participants’ cardiorespiratory responses at maximal effort. Based on the results obtained from the first maximal exercise test, participants performed a second test, which consisted of several bouts of arm cranking at high intensity, i.e. 85–95% of their peak heart rate, followed by a maximal exercise test. Both tests were performed at the clinical physiological laboratory at Sunnaas Rehabilitation Hospital.

### Maximal arm cranking test

Participant performed a five minute warm-up with a work load of 20 watts and a crank rate of 60 revolutions per minute (RPM) on an electrically braked arm cycle ergometer (LODE, Groningen, The Netherlands). During the maximal exercise test, workload was increased individually (5–30 Watt) every third minute, until exhaustion. Oxygen uptake (VO_2_; L/min and ml/kg/min), carbon dioxide production (VCO_2_; L/min), respiratory exchange ratio (RER) and pulmonary ventilation (VE; L/min) were continuously measured by a computerized standard open-circuit technique breath-by-breath spirometer (V_MAX_ Encore 229D, CareFusion Cooperation, San Diego, CA, USA). Heart rate (HR) was measured continuously with a HR monitor (Polar M400, Kempele, Finland). Results for cardiorespiratory fitness level (peak VO_2_) and peak HR are presented in Table 1. The highest achieved HR and the highest achieved mean VO_2_ over 30 s was considered as respectively peak HR and peak VO_2_.

**Table 1:** Demographics and injury-specific characteristics (mean ± SD).

Demographics	SCI	Control
	*n* = 6	*n* = 6
Gender (M/F)	5/1	5/1
Age (years)	51.0 (13.8)	51.2 (13.5)
Body height (meter)	1.84 (0.08)	1.78 (0.13)
Body mass (kg)	82.3 (8.3)	84.3 (13.8)
BMI	24.6 (3.2)	26.5 (1.6)
*Injury-specific characteristics*		
Time since injury (years)	20.5 (19.9)	—
Level of injury	Thoracic 2– 8	—
AIS	A	—

*SD* standard deviation, *SCI* Spinal Cord Injury, *m* meter, *kg* kilogram, *BMI* Body Mass Index, *AIS* American spinal cord association Injury Scale.

### High intensity interval test

After a five-minute warm-up, participants performed high intensity interval exercise on the arm crank ergometer (3 bouts of 4 min at an intensity of 85–95% of the peak heart rate (HR_peak_)), with an active resting period between intervals (at 70% of HR_peak_). During the first three bouts, participants exercised at a predetermined constant workload, estimated from the maximal arm cranking test (at 85% HR_peak_). In some cases, the workload was adjusted to keep exercise intensity between 85–95% HR_peak_. After the third interval and a three minute rest period, the workload during the maximal exercise test was increased every minute, until exhaustion.

All subjects were asked to refrain from alcohol, exercise or strenuous physical activity 24 h prior to the exercise test and not to drink coffee/tea or eat in the hour preceding the test and until the final blood sample was collected (24 h after the second test).

**Fig. 1:**
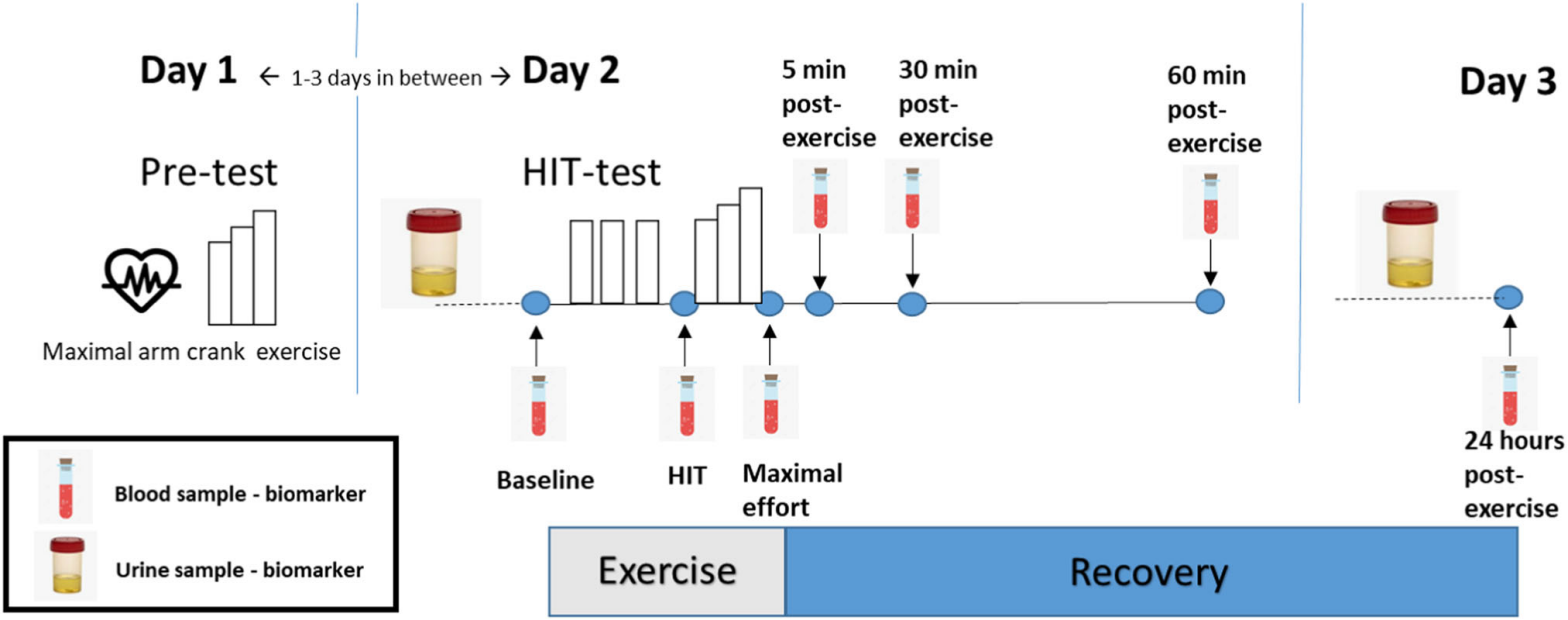
Time line for the research protocol, including blood and urine samples taken before, during and after the exercise bout at high intensity (HIT)/maximal effort.

Figure 1 shows the whole research protocol in a time line, including biomarkers.

### Outcome measures

The primary outcome measures were urine and blood biomarkers for oxidative stress and antioxidants. Morning urine was collected of all participants at the day of the second exercise test (pre-exercise) and the following day (post-exercise). Urine and plasma aliquots were frozen down to −20 °C before stored in liquid nitrogen and −80 °C. However, for vitamin C analysis an equal volume of plasma were acidified with 10% meta-phosphoric acid (MPA) and then stored in −80 °C [16].

Blood samples were collected at six time points, on the day of the second exercise test; before the test (baseline), after high intensity exercise (1–2 min after third interval), after maximal effort (1–2 min after termination), at 5 min post-exercise, 30 min post-exercise, 60 min post-exercise and 24 h post exercise [17].

Measurement of plasma vitamin C (ascorbate (AA), dehydroascorbate (DHAA) and total AA (TAA)) and DHAA/TAA ratio (a plasma marker of oxidative stress) was performed as described by Karlsen et al. [16].

Measurement of plasma carotenoids (lutein, zeaxanthin, β-kryptoxanthin, α-carotene, β-carotene, and lycopene), vitamin E (α-tocopherol) were performed as described by Bastani et al. 2012 [7].

### Measurement of plasma thiols

Amino acids, total glutathione (tGSH), total cysteine (tCys) and total homocysteine (tHcy) were measured at all six time points using plasma samples from blood collection. Plasma were stored at −80 °C. After thawing and vortexing, 10 µL of plasma, calibration standards, QC samples, and blank matrix (water) were added to 96 well plates. Internal standard working solution (10 µL) and 10 μL dithioerythritol (DTE) [18] was added to all wells. Plates were shaked on a plate shaker and left at room temperature for 15 min. 5-Sulfosalicylic acid (5-SSA) [10%] (10 µL) was added to all wells to precipitate the plasma proteins. The solution in the wells were mixed on a plate shaker and centrifuged at 4000 rpm for 15 min. Supernatants (15 µL) from the extraction plate were diluted in 135 μl mobile phase A in a shallow 96 well analysis plate. Aliquots (15 µL) were injected into the LC/MS/MS system for analysis. The injector was temperature regulated at 6 °C.

Chromatographic separation was performed on Phenomenex Kinetex Core Shell C18 (100 × 4.6 mm, 2.6 μm) and temperature regulated at 30 °C. The mobile phases were (A) water with 0.5% formic acid, 0.3% heptafluorobutyric acid (HFBA) and (B) acetonitrile at a flow rate of 0.8 ml/min. The separation was achieved with a linear gradient from 100% (A) to 80% (A) over 6 min and 100% (A) to 40% (A) over 10.5 min, followed by a linear gradient back to 100% (A) over 12 min. The injection volume was 15 µL. Then each amino acid was identified by MS, corresponding to each particular internal standard. The concentration of each amino acid was determined from the ratio of analyte peak area / internal standard peak area against a linear multiple point calibration curve. For calibration and quality assurance, we used NIST standard reference material, ensuring alignment with established standards and facilitating result accuracy and comparability. In the case of amino acids, we compared internal quality controls against Erndim reference controls, further validating the accuracy and reliability of our measurements. [19]

### Measurement of urinary F2-Isoprostane and creatinine

The determination of the isoprostane 8-epi-PGF2α a biomarker for lipid peroxidation was performed by liquid chromatography–mass spectrometry (LC/MS/MS) [18].The creatinine level in urine used for the normalization of 8-epi-PGF2 α concentration (8-epi-PGF2 α ng/g creatinine). 8-Oxo-2’-deoxyguanosine (8-oxo-dG) is a major product of DNA oxidation and level of 8-oxo-dG in urine is a measurement of oxidative stress. Urine samples (1 mL) were treated with internal standards (8-oxo-dG-15N^5^) and (Cr-d3) applied to solid phase extraction (SPE) clean-up columns (100 mg C18 EC) from Isolute Biotage (Uppsala, Sweden) preconditioned with 1 mL methanol and 1 ml water. Bound samples were eluted with 1 ml methanol: water (1:4). Eluates were dried gently with hot nitrogen, and the dried residues were dissolved in 200 μL water and 15 ml aliquots were injected for LC/MS/MS analysis. Creatinine level in urine were used for normalization of 8-oxo-dG concentration (8-oxo-dG ng/g creatinine).

#### Measurements of serum CRP, albumin and creatinine

Serum CRP, creatinine and albumin were measured routinely using dry-slide technology with Vitros 250 Chemistry System by Ortho Clinical Diagnostics (Johnson & Johnson, Auckland, New Zealand).

#### Statistical analyses

The sample size is based on previous literature evaluating antioxidant status among persons with spinal cord injury and able-bodied after long term and acute exercise [20–22]. Sample size calculation was not performed as (to the best of our knowledge) our outcome measures, type of exercise and study design are different from previous studies.

The statistical analyses were performed with SPSS version 28.0 (Chicago, IL). Data are reported as mean (SD or SEM) unless otherwise stated. The concentration of blood plasma reduces during physical exercise [23], depending on the intensity. Changes in Albumin (g/L) have been shown to reflect changes in bloodplasma concentration [24]. Since healthy subjects can perform exercise at a higher workload compared to persons with SCI, changes in albumin at each timepoint was used to adjust outcome biomarkers for changes in bloodplasma concentration. Blood biomarkers for oxidative stress and antioxidants during high intensity training (HIT) and at maximal effort are presented as adjusted mean values (SD) from baseline, including the percentage change (from baseline to HIT and maximal effort) among participant with SCI and control group. To evaluate if changes in blood biomarkers from baseline to HIT (and from baseline to maximal effort), were different between the SCI and control group, an Independent Samples T-test was performed to compare the % change in these two groups. Changes in 8oxo-dG (ng/g) and 8-Iso-pgf2α (ng/g), from pre-exercise to post-exercise between the SCI and control group were compared with Independent Samples T-test. We considered *p* values less than 0.05 to indicate statistical significance. Cohen’s d was used to describe effect size of the differences in both urine- and blood-biomarkers between the SCI and control group.

## Results

Participants’ spinal cord injury-specific characteristics and demographics for participants with SCI and (matched) healthy controls are described in Table 1.

All participants performed the pre-test at maximal effort, the interval exercise test at 85–95% of their peak HR, and ended the interval session at maximal effort. In Table 2, cardiorespiratory responses during these test are described. During the second exercise test, the intensity (at HIT and maximal effort) is described as the percentage of peak HR, which is calculated from peak HR from the pre-test. Table 2 show high levels of percentage peak HF, [LA^-^] and RER at maximal effort and a high percentage of peak HR during HIT, indicating that participants in both groups were exercising at adequate intensity levels. Oxygen uptake, RER and [LA^-^] were not measured during HIT exercise.

**Table 2:** Cardiorespiratory responses (mean (SD)) during pre-test, high intensity training (HIT) and maximal effort during arm cranking in the SCI and control group.

	Pre-test		HIT		Maximal effort	
	SCI (*n* = 6)	control (*n* = 6)	SCI (*n* = 6)	control (*n* = 6)	SCI (*n* = 6)	control (*n* = 6)
Load (watt)	88 (40)	114 (22)	70 (34)	92 (20)	84 (45)	107 (18)
Peak HR (beats/min)	157 (28)	170 (15)	143 (30)	156 (23)	159 (31)	168 (24)
Intensity (%peak HF)	100%	100%	91% (6%)	92% (6%)	101% (9%)	99% (8%)
Peak VO_2_ (L/min)	1.58 0.58)	2.33 (0.53)	—	—	1.49 (0.56)	2.57 (0.59)
Peak VO_2_ (ml/kg/min)	19.6 (9.2)	27.6 (5.4)	—	—	18.4 (8.7)	30.5 (5.9)
[L^-^] (mMol/L)	9.91(2.14)	11.66 (1.38)	—	—	8.67 (3.21)	11.73 (1.26)
RER	1.40 (0.11)	1.35 (0.06)	—	—	1.36 (0.12)	1.22 (0.08)

*SD* standard deviation, *SCI* Spinal Cord Injury, *AIS* American spinal cord association Injury Scale, *HR* heart rate, *VO*_*2*_ oxygen uptake, *min* minute, *ml* milliliter, *[LA-]* blood lactate, *RER* Respiratory Exchange Ratio.

Tables 3 and 4 show several biomarkers, including carotenoids at rest (baseline), during high intensity exercise (Table 3) and at maximal effort (Table 4).

**Table 3:** Mean values (SD) of biomarkers (blood) for oxidative stress and antioxidants at baseline and during high intensity training (HIT), including change (%) in the SCI and control group.

Biomarker	Baseline mean (SD)		HIT^a^ mean (SD)		% change baseline – HIT^a^ mean (SD)			
	SCI (*n* = 6)	control (*n* = 6)	SCI (*n* = 6)	control (*n* = 6)	SCI (*n* = 6)	control (*n* = 6)	*p*-value^b^	Effect size^c^
Albumin (g/L)	39.5 (2.3)	43 (2.8)^d^	42.7 (3.9)	50.1 (3.7)	+7.1 (4)	+14.1 (3)	0.008^d^	−1.92
Creatinine (µmol/L)	59.3 (134)	75.7 (7.1)^d^	55.9 (10.4)	69.9 (9.1)	−4.8 (5.7)	−7.8 (5.6)	0.383	0.53
TAA (µmol/L)	32.1 (5,1)	33.8 (11.0)	31.4 (6.3)	30.3 (8.7)	−3.4 (10.1)	−11.5 (11.5)	0.22	0.75
DHAA/TAA ratio	0.23 (0.04)	0.20 (0.12)	0.23 (0.05)	0.28 (0.17)	−1.4 (27.5)	+15.8 (43)	0.43	−0.48
α-tocopherol (µmol/L)	25.5 (7.07)	27.9 (3.8)	26.6 (6.2)	26.9 (5.2)	+4.4 (13.0)	−5.7 (18.7)	0.30	0.63
*Amino acids*								
Gluthathione (µM)	3.73 (0.81)	4.58 (1.16)	2.94 (0.85)	3.68 (0.71)	−37.8 (62.6)	−25.1 (27.9)	0.30	−0.26
Valine (µmol/L)	73.7 (18.9)	94.8 (40.8)	67.8 (12.3)	82.8 (45.9)	−8.0 (12.6)	−19.6 (20.2)	0.26	0.69
Isoleucine (µmol/L)	39.8 (15.5)	33.8 (17.7)	29.7 (11.8)	27.9 (12.7)	−35.1 (24.5)	−24.7. (33.0)	0.55	−0.34
Leucine (µmol/L)	71.6 (17.6)	75.5 (25.5)	58.2 (14.9)	63.2 (24.5)	−23.3 (11.5)	−22.2 (20.0)	0.91	−0.06
Homocysteine (µM)	16.46 (6.88)	12.81 (5.16)	15.55 (8.78)	12.32 (5.26)	−25.3 (58.3)	−4.2 (15.7)	0.13	−0.49
Cysteine (µM)	580.3 (90.5)	688.4 (407.0)	504.2 (92.1)	508.7 (167.0)	−18.3 (30.8)	−28.7 (32.6)	0.95	0.33
*Carotenoids*								
Lycopene (nmol/L)	0.60 (0.39)	0.80 (0.45)	0.54 (0.36)	0.74 (0.38)	−12.0 (7.7)	−5.0 (12.6)	0.27	−0.67
α-carotene (nmol/L)	0.08 (0.05)	0.22 (0.09)^d^	0.07 (0.05)	0.21 (0.11)	−9.0 (5.9)	−6.4 (13.0)	0.66	−0.52
β-carotene (nmol/L)	0.29 (0.16)	0.80 (0.45)^d^	0.27 (0.16)	0.78 (0.43)	−9.3 (5.3)	−4.7 (11.7)	0.40	−0.47
β-Cryptoxanthin (nmol/L)	0.10 (0.06)	0.23 (0.12)^d^	0.09 (0.05)	0.23 (0.14)	−2.3 (4.5)	−5.1 (11.3)	0.58	0.33
Lutein (µmol/L)	0.17 (0.04)	0.29 (0.12)	0.17 (0.04)	0.28 (0.14)	+0,2 (5.0)	−5.1 (10.1)	0.28	0.67
Zeaxanthin (µmol/L)	0.05 (0.01)	0.08 (0.03)	0.05 (0.01)	0.09 (0.03)	−0.4 (4.5)	−4.9 (11.0)	0.37	0.54

*HIT* High Intensity Training, *SD* standard deviation, *SCI* Spinal Cord Injury, *µmol/L* micromole per liter, *nmol/L* nanomoles per liter, *TAA* total ascorbic acid, *DHAA/TAA* dehydroascorbic acid/total ascorbic acid.

^a^All individual measured values have been adjusted for differences in blood plasma volume (by correcting for the change in albumin).

^b^Significance level describing the difference between the SCI and control group.

^**c**^Cohens’d effect size for the comparison between the two means (SCI and control).

^d^Significant difference between the SCI and the control group (*p* < 0.05).

**Table 4:** Biomarkers for oxidative stress and antioxidants at baseline and during arm cranking at maximal effort including change (%) in the SCI and control group.

Biomarker	Baseline mean (SD)		Maximal effort^a^ mean (SD)		% change baseline – maximal effort^a^ mean (SD)			
	SCI (*n* = 6)	control (*n* = 6)	SCI (*n* = 6)	control (*n* = 6)	SCI (*n* = 6)	control (*n* = 6)	*p*-value^b^	Effect size^c^
Albumin (g/L)	39.5 (2.3)	43 (2.8)d	45.5 (4.5)	53.0 (3.4)	+12.8 (5.4)	+18.8 (2.7)	0.035d	−1.40
Creatinine (µmol/L)	59.3 (13.4)	75.7 (7.1)^d^	54.7 (11.2)	70.3 (9.0)	−8.0 (7.7)	−8.1 (7.5)	0.97	0.02
TAA (µmol/L)	32.1 (5.1)	33.8 (11.0)	29.4 (4.0)	28.1 (8.0)	−9.8 (13.1)	−21.6 (20.3)	0.26	0.69
DHAA/TAA ratio	0.23 (0.04)	0.20 (0.12)	0.25 (0.11)	0.28 (0.17)	−9.2 (62.2)	+43.8 (22.8)	0.078	−1.13
α-tocopherol (µmol/L)	25.5 (7.07)	27.9 (3.8)	23.2 (6.4)	25.3 (5.1)	−10.3 (17.3)	−12.3 (17.4)	0.84	0.12
*Amino acids*								
Gluthathione (µM)	3.73 (0.81)	4.58 (1.16)	2.86 (0.79)	3.86 (0.54)	−39.0 (47.6)	−18.3 (25.9)	0.26	−0.54
Valine (µmol/L)	73.7 (18.9)	94.8 (40.8)	60.8 (17.4)	72.3 (28.7)	−25.0 (26.8)	−31.5 (19.4)	0.64	0.28
Isoleucine (µmol/L)	39.8 (15.5)	33.8 (17.7)	29.0 (12.2)	23.0 (8.8)	−42.7 (25.6)	−44.3 (34.9)	0.93	0.05
Leucine (µmol/L)	71.6 (17.6)	75.5 (25.5)	53.8 (14.4)	56.1 (17,2)	−34.7 (18.6)	−34.0 (13.3)	0.95	−0.04
Homocysteine (µM)	16.46 (6.88)	12.81 (5.16)	15.26 (7.0)	12.43 (5.1)	−10.1 (25.9)	−3.3 (9.0)	0.07	−0.36
Cysteine (µM)	580.3 (90.5)	688.4 (407.0)	496.3 (111.0)	493.6 (144.4)	−20.1 (24.3)	−33.0 (40.9)	0.44	0.38
*Carotenoids*								
Lycopene (nmol/L)	0.60 (0.39)	0.80 (0.45)	0.52 (0.32)	0,72 (0.37)	−15.1 (10.7)	−8.5 (9.8)	0.29	−0.64
α-carotene (nmol/L)	0.08 (0.05)	0.22 (0.09)^d^	0.07 (0.05)	0.20 (0.09)	−12.8 (9.1)	−6.9 (8.0)	0.26	−0.65
β-carotene (nmol/L)	0.29 (0.16)	0.80 (0.45)^d^	0.26 (0.15)	0.74 (0.37)	−12.3 (8.8)	−7.3 (8.4)	0.34	−0.62
β-Cryptoxanthin (nmol/L)	0.10 (0.06)	0.23 (0.12)^d^	0.09 (0.05)	0.22 (0.12)	−7.4 (4.4)	−7.5 (6.5)	0.96	0.03
Lutein (µmol/L)	0.17 (0.04)	0.29 (0.12)	0.16 (0.03)	0.24 (0.11)	−5.2 (5.4)	−24.7 (28.5)	0.13	0.95
Zeaxanthin (µmol/L)	0.05 (0.01)	0.08 (0.03)	0.05 (0.01)	0.08 (0.03)	−4.6 (4.2)	−7.6 (5.2)	0.29	0.65

*SD* standard deviation, *SCI* Spinal Cord Injury, *µmol/L* micromole per liter, *nmol/L* nanomoles per liter, *TAA* total ascorbic acid, *DHAA/TAA* dehydroascorbic acid/total ascorbic acid.

^a^All individual measured values have been adjusted for differences in blood plasma volume (by correcting for the change in albumin).

^b^Significance level describing the difference between the SCI and control group.

^**c**^Cohens’d effect size for the comparison between the two means (SCI and control).

^d^Significant difference between the SCI and the control group (*p* < 0.05).

Participants with SCI had significant lower baseline blood levels of α-carotene (*p* < 0.001), β-carotene (*p* = 0.01), β-cryptoxanthin (*p* = 0.04), albumin (*p* = 0.04) and creatinine (*p* = 0.03) compared to controls (Table 3). During high intensity exercise, healthy controls had a significant higher percentage increase (from baseline) in albumin, compared to SCI participants. No other between-group difference in change of biomarkers from baseline to HIT were found.

Nine out of twelve participants had normal CRP levels (< 10 mg/L), at each timepoints. Three of the participants with SCI showed slightly increased levels of CRP (> 10 mg/L) at baseline. These increased levels remained stable (between 11 and 22 mg/L) at all timepoints.

From baseline to HIT, blood concentrations of both amino acids and carotenoids changed similar in the SCI and control group (i.e. amino acids: 8–38% decrease in the SCI cases and 4–29% decrease in control cases; carotenoids: decreased by 0–12% in SCI cases and 5–6% in control cases).

During exercise at maximal effort, healthy controls had a significant higher percentage increase (from baseline) in albumin (Table 4) compared to SCI cases. At maximal effort, none of the other biomarkers had changed (%) significantly different between the SCI and control group (i.e. amino acids decreased by 10–43% in the SCI cases and 3–44% in control cases; and the concentration carotenoids decreased by 5–15% in SCI cases and 7–25% in control cases).

**Fig. 2:**
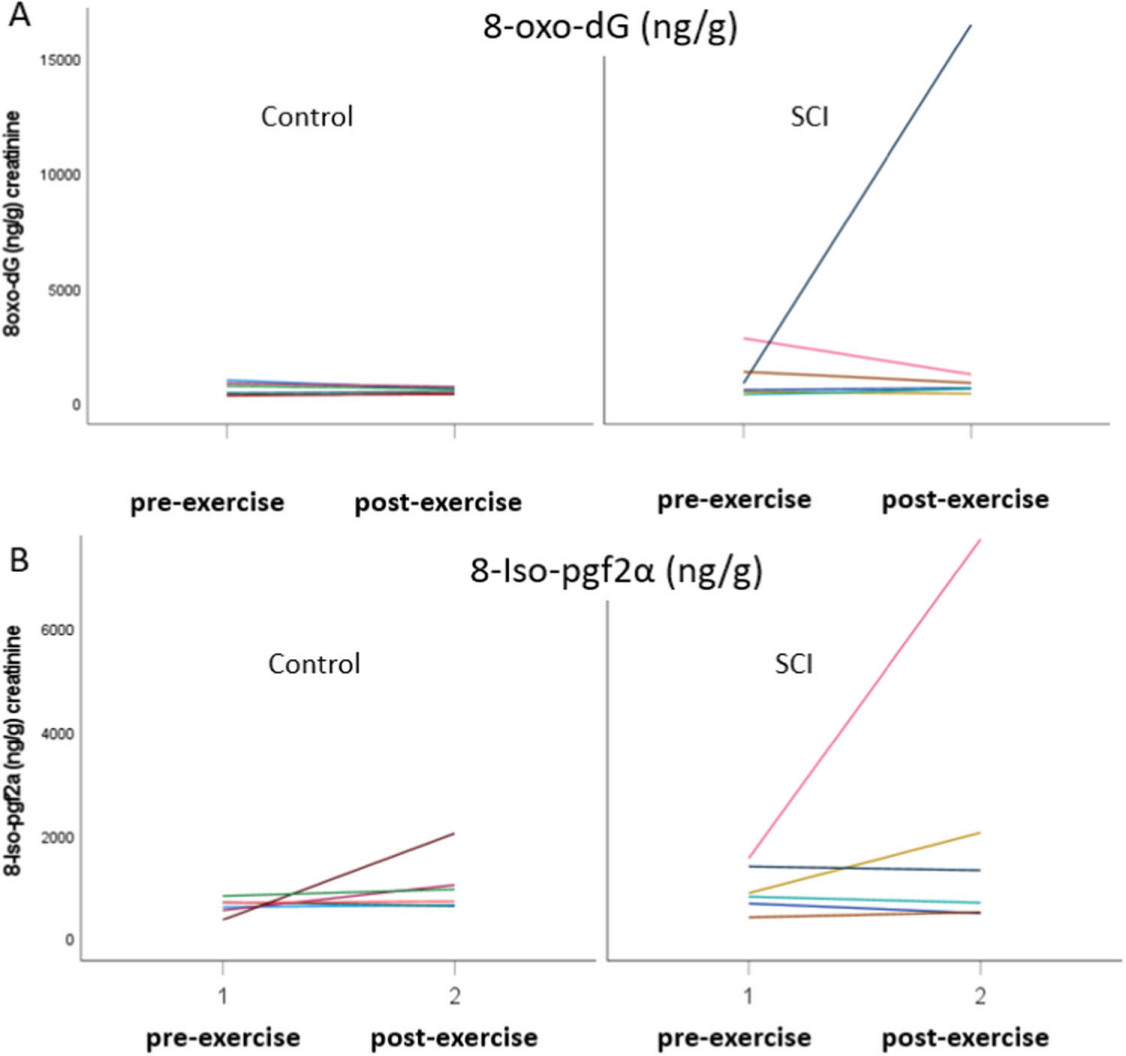
Urinary biomarkers for oxidative stress among participants with spinal cord injury (SCI) and able-bodied. **A** 8-Oxo-2’-deoxyguanosine (8-oxo-dG); **B** isoprostane 8-epi-PGF2α.

Urine biomarkers of oxidative stress were not significant different in participants with a SCI and able-bodied at pre-test and posttest. The percentage change from pre- to posttest, in 8oxo-dG (ng/g) and 8-Iso-pgf2α (ng/g) concentration, was similar in both groups (Table 5). However, individual variation was higher among participants with a SCI, as shown in Fig. 2 A, B.

**Table 5:** Urine concentrations for biomarkers for oxidative stress levels pre-exercise and post-exercise (day after).

Biomarker	pre-exercise (SD)		*p*	post-exercise (SD)		*p*	change pre-post (95% CI)		difference (95% CI)	*p*	effect size
	SCI (*n* = 6)	con (*n* = 6)		SCI (*n* = 6)	con (*n* = 6)		SCI (*n* = 6)	con (*n* = 6)	SCI/con		
8oxo-dG (ng/g)	1068 (922)	609 (299)	0.27	3371 (6404)	533 (125)	0.30	2303 (−4558–9165)	−77 (−288–134)	2380 (−4481–9241)	0.39	0.51
8-Iso-pgf2α (ng/g)	944 (433)	615 (159)	0.11	2120 (2798)	994 (529)	0.36	1175 (−1441–3793)	379 (−314–1072)	797 (−1814–3408)	0.47	0.44

*SD* standard deviation, *SCI* Spinal Cord Injury, *CI* Confidence Interval, *ng/g* nanogram per gram.

**Fig. 3:**
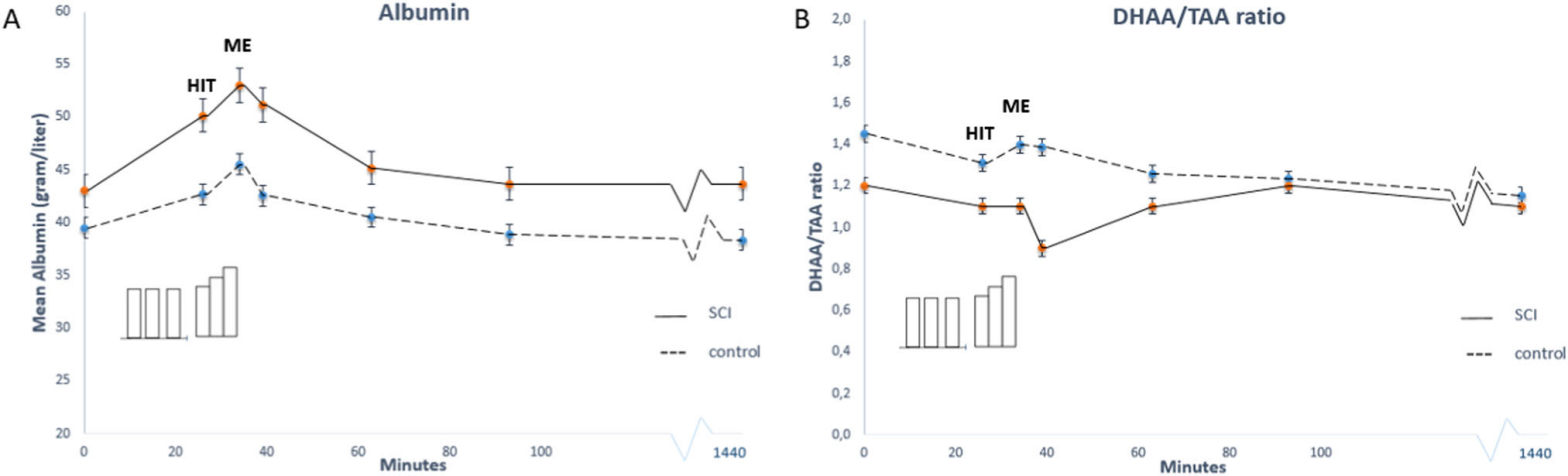
Mean albumin and DHAA/TAA ratio at seven different time points during the 24 h (1440 min) protocol, for both groups. **A** Albumin; **B** DHAA/TAA ratio.

Panelplots showing mean albumin and mean DHAA/ TAA ratio (a plasma marker of oxidative stress) at seven different timepoints during the 24 h (1440 min) protocol, for both groups (Fig. 3A, B).

## Discussion

In the present study, we investigated several biomarkers of oxidative stress and antioxidants at rest, during and after vigorous exercise among persons with SCI matched with able-bodied controls. Our study indicate that paraplegics (AIS A, thoracic injury level) have lower plasma levels of antioxidants and creatine, but similar antioxidative changes in response to HIT and maximal effort exercise compared to matched controls. However, individual variation were higher in the SCI group compared to controls.

A study by Parker et al. among 14 non-smoking, sedentary healthy adult males, found that moderate-to-high-intensity exercise significantly elevated the biological antioxidant potential in plasma (most likely driven by uric acid), suggesting a counter-reaction to exercise-induced ROS [25]. The present study shows a similar change in redox status in response to high intensity exercise, both in able-bodied and in the paraplegic participants. Strenuous exercise increases the oxidative stress in muscles by overproduction of free radicals in humans [9, 25]. Athletes and able-bodied persons that exercise regularly show improved antioxidant response after graded exercise compared to sedentary controls. Van Duijnhoven et al. (2010) found a moderate correlation between lower oxidative stress baseline values and higher aerobic fitness amongst SCI subjects [22]. It has been demonstrated similar findings among tetraplegic rugby players compared to a sedentary matched control group [26]. Physical exercise may affect the level of blood plasma concentrations, especially during workouts with increasing intensity. This could influence biomarkers of oxidative stress and antioxidant levelse in able-bodied and SCI individuals [27]. Albumin has shown to be a good marker of changes in blood plasma concentration [24]. In our study able-bodied individuals produced more muscular work during arm-cranking exercise than individuals with SCI. During exercise, a higher amount of muscular work is reflected by a higher concentration of albumin in blood plasma, indicating larger hemoconcentration with increasing workload. Higher amount of work produced by the able-bodied control group may therefore explain the increased change in albumin levels compared to the SCI group (Table 3).

Changes in biomarkers of oxidative stress and antioxidants in response to high-intensity exercise was, however, similar among participants with SCI and controls, suggesting a normal physiological response. However, we observed large individual changes in 8oxo-dG (ng/g) and 8-Iso-pgf2α (ng/g) (from pre- to post test) among SCI participants compared to able bodied controls. This could indicate the presence of other injury-related factors, such as chronic lowgrade inflammation, affecting changes in the antioxidative response to vigorous exercise demonstrated in our SCI samples. Lower baseline levels in albumin were found among the participants with SCI compared to the matched controls. Hypoalbuminia is associated with inflammation and previously described in SCI [28]. This might also explain the elevated CRP levels ≥10 (ng/ml) seen in some of the SCI participants.

At rest, the participants with SCI had significantly lower levels of α-carotene and β-carotene compared to able-bodied participants, which is similar to our previous findings [7]. We found that SCI patients had a significantly reduced level of plasma antioxidants and increased markers of oxidative stress in the acute phase (1 month after injury) compared to able-bodied controls. Although there were some improvement over time, the SCI subjects still had a significantly lower level of antioxidants in plasma up to one year after injury. Lower levels of exogenous antioxidants may be a combination of poor diet, low grade inflammation and physiological responses to physical activity as discussed. A healthy diet based on the national recommendations provides important antioxidants and can potentially upregulate the endogenous antioxidative capacity [29]. The most important antioxidants in our diet include vitamin E, vitamin C, carotenoids and flavonoids. Studies that have examined the nutritional intake after SCI indicate that people with SCI tend to deviate from the nutritional recommendations [30, 31], e.g. they tend to eat less than the recommended intake of antioxidant vitamins (C, E and A), have lower plasma concentrations of carotenoids and consume higher amounts of fat compared to a general population.

Our study disclosed results that needs further investigation. More specifically, a larger effect study with a combined nutritional and exercise intervention would be of interest. Our results indicate that the antioxidative response after aerobic exercise at high intensity is highly individual in the SCI population. This highlights the need of a individually adapted exercise programs, including nutritional strategies, in this patient population. More specifically, the effect of a nutritional intervention combined with a high intensity exercise program on oxidative stress *over time* remains to be explored.

### Methodological considerations

Despite the low number of participants, this study was robustly designed and the study subjects were carefully monitored under standardized conditions. By performing a maximal arm crank pre-test, participants exercised at the intended intensity, being a strength in this study. Astorino et al. showed that able-bodied participants needed a verification test to validate appearance of a ‘true’ peak VO_2_ during arm cycling [32]. Therefore the pre-test in our study might have underestimated the peak VO_2_ in the able-bodied participants and consequently they possibly exercised at lower intensity as intended.

Another weakness in the study is that dietary intake of antioxidants were not examined. A difference in dietery intake could have explained some of the differences in exogenous antioxidant biomarkers between the SCI and the control group.

## Conclusion

The participants with SCI in this study showed a similar change in oxidative stress levels and antioxidant levels in response to high-intensiv exercise compared to matched able bodied participants. However the individual variations of oxidative stress levels were high among SCI cases and they have lower plasma levels of exogenous antioxidants at rest. This indicates that the antioxidative capacity after aerobic exercise at high intensity is highly individual in the SCI population. This emphasizes the need to investigate effects of individually adapted exercise programs, combined with high attention to recovery strategies, including nutrition interventions.

## Supplementary information

AJ-checlist

## Data Availability

Additional data are available from the corresponding author on reasonable request.
